# Effects of Various Phytochemicals on Indoleamine 2,3-Dioxygenase 1 Activity: Galanal Is a Novel, Competitive Inhibitor of the Enzyme

**DOI:** 10.1371/journal.pone.0088789

**Published:** 2014-02-12

**Authors:** Rie Yamamoto, Yasuko Yamamoto, Shinjiro Imai, Ryuta Fukutomi, Yoshio Ozawa, Masako Abe, Yushi Matuo, Kuniaki Saito

**Affiliations:** 1 Human Health Sciences, Graduate School of Medicine and Faculty of Medicine, Kyoto University, Kyoto-City, Kyoto, Japan; 2 Graduate School of Nutrition and Environmental Sciences, University of Shizuoka, Shizuoka-City, Shizuoka, Japan; 3 Health Care Research Center, Nisshin Pharma Inc., Chiyoda-ku, Tokyo, Japan; 4 Health and Nutrition, Takasaki University of Health and Welfare, Takasaki-City, Gunma, Japan; Tokyo Metropolitan Institute of Medical Science, Japan

## Abstract

Indoleamine 2,3-dioxygenase (IDO) 1, that catalyzes the first and rate-limiting step in the degradation of L-tryptophan, has an important immunomodulatory function. The activity of IDO1 increases in various inflammatory diseases, including tumors, autoimmune diseases, and different kinds of inflammation. We evaluated the suppressive effect of plant extracts or phytochemicals on IDO1 induction and activity; sixteen kinds of plants extracts and fourteen kinds of phytochemicals were examined. As a result, the methanol extracts of Myoga flower buds, which are traditional Japanese foods, and labdane-type diterpene galanal derived from Myoga flowers significantly suppressed IDO1 activity. The Lineweaver-Burk plot analysis indicated that galanal is a competitive inhibitor. Galanal attenuated L-kynurenine formation with an IC_50_ value of 7.7 µM in the assay system using recombinant human IDO1, and an IC_50_ value of 45 nM in the cell-based assay. Further, mechanistic analysis revealed that galanal interfered with the transcriptional function of the nuclear factor-κB and the interferon-γ signaling pathway. These effects of galanal are important for immune response. Because the inhibitory effect of galanal on IDO1 activity was stronger than that of 1-methyl tryptophan, a tryptophan analog, galanal may have great potential as the novel drug for various immune-related diseases.

## Introduction

Indoleamine 2,3-dioxygenase 1 (IDO1, EC 1.13.11.42) is the first and rate-limiting enzyme in the tryptophan-kynurenine pathway and degrades the essential amino acid L-tryptophan (L-Trp). IDO1 is induced by interferon-γ (IFN-γ)-mediated effects of the signal transducer and activator of transcription 1α (STAT1-α), and interferon regulatory factor 1 (IRF-1) [1]. The induction of IDO1 can also be mediated through an IFN-γ-independent mechanism. The induction of IDO1 by lipopolysaccharide (LPS) is regulated by the p38 mitogen-activated protein kinase (MAPK) pathway and nuclear factor-κB (NF-κB) [2] [3].

The metabolism of L-Trp via IDO1 is accompanied by the production of a series of immunoregulatory metabolites, collectively known as “kynurenines,” which can suppresses the proliferation and differentiation of effector T cells [4], and markedly enhance the suppressor activity of regulatory T cells [5]. As a result, IDO1 controls and fine-tunes both innate and adaptive immune responses [6] under a variety of conditions, including pregnancy[7], transplantation[8], infection [9], chronic inflammation [10], autoimmunity [11], neoplasia, and depression[12]. Owing to the outstanding immune-modulate properties of IDO1, IDO1 inhibitors have been searched for in many fields, to control various inflammatory diseases. Thus, it is hoped that the inhibitor of IDO1 becomes the new therapeutic target for drugs corresponding to various inflammatory diseases [13] [14].

Previous researches have given direct evidence of the crucial role of natural products from plants, animals, and micro-organisms as potential sources of various modern pharmaceuticals. Currently, phytochemical research is being considered an effective approach in the discovery of novel chemical entities, with potential as drug leads. Previous reports have shown that some food compounds such as epigallocatechin gallate (EGCg; CID 65064) and curcumin (CID 969516) inhibit the induction of IDO1[15] [16]. Therefore, we extracted various compounds from traditional Japanese foods and plants. The purpose of this study was to find a novel effective inhibitor of IDO1 from food and plant compounds. We examined the inhibitory effects of fourteen kinds of plant extracts and sixteen kinds of phytochemicals on the induction of IDO1. Among these compounds, we found that galanal (CID 3050416) isolated from the methanol extract of Myoga flower buds was the most effective inhibitor of IDO1.

## Materials and Methods

### Materials

Docosahexaenoic acid (DHA, (22∶6), CID 445580), eicosapentaenoic acid (EPA, (20∶5), CID 446284), epigallocatechin gallate (EGCG), L-Trp, L-kynurenine (L-Kyn) and recombinant human IFN-γ (rhIFN-γ) were purchased from WAKO Chemical (Tokyo, Japan). DHA and EPA were dissolved in 100% ethanol and each 20 mM solution was prepared for storing at −30°C. The purification of phytochemicals used, except EGCG from plant extracts, and the preparation of plant extracts used were conducted using the same methods as described in previous reports [17].

### Cell culture

Human acute leukemic cells, THP-1, and Human embryonic kidney, HEK293, were maintained in RPMI-1640 or DMEM medium supplemented with 10% FCS, at 37°C in a humid atmosphere of 5% CO_2_. Cells (1×10^6^) were treated with phytochemicals (10 µM) or plant extracts (30 µg/ml), and LPS (50 ng/ml) for 24 hrs.

### Measurement of L-Kyn

L-Kyn in each conditioned medium was measured by the method using high-performance liquid chromatography (HPLC) with a spectrophotometric detector (SHIMADZU, Prominence UFLC), as described in our previous reports [18] [19].

### Expression and purification of recombinant IDO1

The human IDO1 cDNA was expressed in E. coli, and purified by a Ni_2_-column by affinity-binding to the N-His-tag of recombinant IDO1, as described in our previous reports [20]. The resultant IDO1 was enzymatically active when assayed using L-Trp as a substrate. Therefore, this purified IDO1 was used for monitoring IDO1 activity. It is stored at −80°C until use.

### Enzyme assay for rIDO1

IDO1 activity was determined by the methylene blue/ascorbate assay as previously described [3]. The reaction mixture contained 50 µl of rIDO and 50 µl of substrate solution. The composition of the substrate solution was 100 mM potassium phosphate buffer (pH 6.5), 50 µM methylene blue, 20 µg of catalase, 50 mM ascorbate, and 0.4 mM L-Trp. After incubating the reaction mixture at 37°C for sixty minutes, samples were acidified with 3% perchloric acid and centrifuged at 7000×g for 10 min at 4°C. The concentrations of the enzymatic products were measured using HPLC. The type of IDO1 inhibition by galanal was determined from the plot of enzyme kinetics.

### RNA Extraction and semi-quantitative analysis of RT-PCR products

The total RNA was extracted from cell lines with ISOGEN (Nippon GENE, Tokyo, Japan) and the RNA concentration was determined spectrophotometrically at 260 nm. Reverse transcription-PCR was performed by using Revetra Ace Kits (TOYOBO, Japan). Primers used in semi-quantitative analysis were as follows-GAPDH: 5′-accacagtccatgccatcac (sense) and 5′-tccaccaccctgttgctgta (antisense); IDO1: 5′-cctgacttatgagaacatggacgt (sense) and 5′-atacaccagaccgtctgatagctg (antisense). Semi-quantitative analysis of RT-PCR products was performed by using NIH ImageJ 1.34 s software and normalized to GAPDH. When the same experiment was repeated three times, the three experiments showed similar results. Therefore, the results obtained from one of the three experiments were shown.

### Determination of mRNA expression levels using quantitative real-time RT-PCR

To analyze the amount of IDO1 mRNA, real-time PCR was performed by the Applied biosystems 7500 real-time qPCR system (Applied Biosystems, Foster City, USA)using the EvaGreen PCR Master Mix (BioRad, Tokyo, Japan). The mRNA level of IDO was normalized to that for β-actin. Primers used in real-time PCR were as follows-IDO1:5′-GCATTTTTCAGTGTTCTTCGCATA-3′ (sense) and 5′-CATACACCAGACCGTCTGATAGCT-3′ (antisense); β-actin:5′-TGCACCACACCTTCTACAATGA-3′ (sense) and 5′-CAGCCTGGATAGCAACGTACAT-3′(antisense).

### Protein phosphorylation

Protein phosphorylation was examined with using PhosphoTracer ELISA kit (Cambridge, UK). Each experiment was performed according to the manufacture protocols.

### Statistical analysis

Values obtained, except the kinetic study, are expressed as means ± SD. Statistically significant differences (p<0.05) were analyzed by one-way ANOVA, followed by post-hoc test with Tukey-test.

The enzymatic assay of IDO1 in the presence or absence of either each plant extract or each phytochemical was performed in duplicate or triplicate; each assay was repeated at least two or three times. Based on the replicate assays of IDO1 activity in the presence or absence of galanal, the average values of the activities obtained in the replicate assays were shown in all Figures. Each value of IDO1 activity in the replicate assays varied within less than 5% of the average value of the activities.

## Results

### Effects of plant extracts and phytochemicals on induction of IDO1

**Figure 1:**
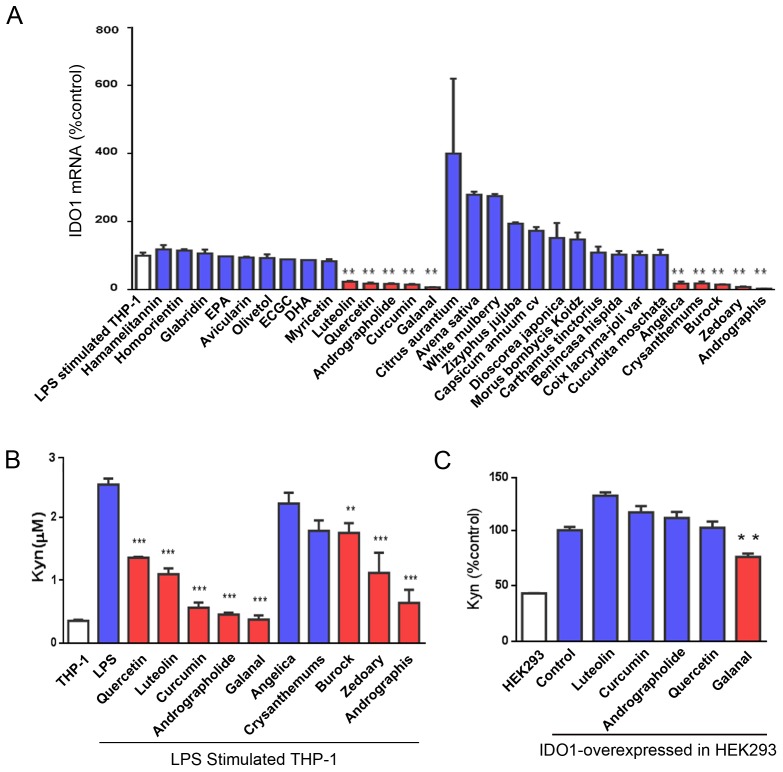
Effects of plant extracts and phytochemicals on IDO1 induction. (A) The expression of IDO1 mRNA in LPS-stimulated THP-1 cells with and without either plant extracts or phytochemicals. THP-1 cells were incubated with LPS (50 ng/ml) and either plant extracts (30 µg/ml each) or phytochemicals (10 µM each). The levels of IDO1 mRNA were measured using RT-PCR. (B) L-Kyn formation from L-Trp in LPS-stimulated THP-1 cells with and without either plant extracts or phytochemicals. L-Kyn formed in the LPS-stimulated cells with each treatment after 24-hr incubation was measured by HPLC. (C) HEK293cells transfected with human IDO1 were incubated with galanal, curcumin, andrographolide, quercetin, or luteolin (5 µM each). The concentration of L-Kyn formed after 48-hr incubation was measured by HPLC. Data are expressed as the mean ± SD (n = 3). ***P<0.001. **P<0.01.

First, the effects of fourteen kinds of plants extracts and sixteen kinds of phytochemicals on IDO1 induction were examined, that is, the expression of IDO1 at its mRNA level was examined in LPS stimulated THP-1 cells treated with and without fourteen kinds of plants extracts and sixteen kinds of phytochemicals (Figure 1A). Among them, five kinds of plants extracts and five kinds of phytochemicals were selected because they had an inhibitory effect on the expression of IDO1 at its mRNA level (Figure 1A). The selected five phytochemicals were luteolin, quercetin, andrographolide, curcumin and galanal. The selected five plant extracts were the extracts of angelica, chrysanthemums, burock, zeodoary, and andrographis. Second, the inhibitory effects of the selected five plant extracts and five phytochemicals on the production of L-Kyn from L-Trp in LPS-stimulated THP-1 cells were examined by measuring the concentration of L-Kyn using HPLC. As shown in Figure 1B, the levels of L-Kyn produced via IDO1 in LPS-stimulated THP-1 cells were reduced extensively by some compounds such as quercetin, luteolin, curcumin, andrographolide and galanal. Thus, andrographolide and galanal present in the extract of Myoga were able to inhibit IDO1 induction. Galanal, especially, had a strong inhibitory effect on IDO1 induction.

Because quercetin, luteolin, curcumin, andrographolide and galanal clearly showed an inhibitory effect on IDO1 induction, we further examined these compounds using IDO1-overexpressed HEK293 cells, to see whether they inhibited the enzymatic activity of IDO1 or not (Figure 1C). Among the five compounds, galanal showed a significant inhibition of the enzymatic activity of IDO1 in IDO1-overexpressed in HEK293 cells.

### Inhibitory effect of galanal on IDO1 activity in different cell species

**Figure 2:**
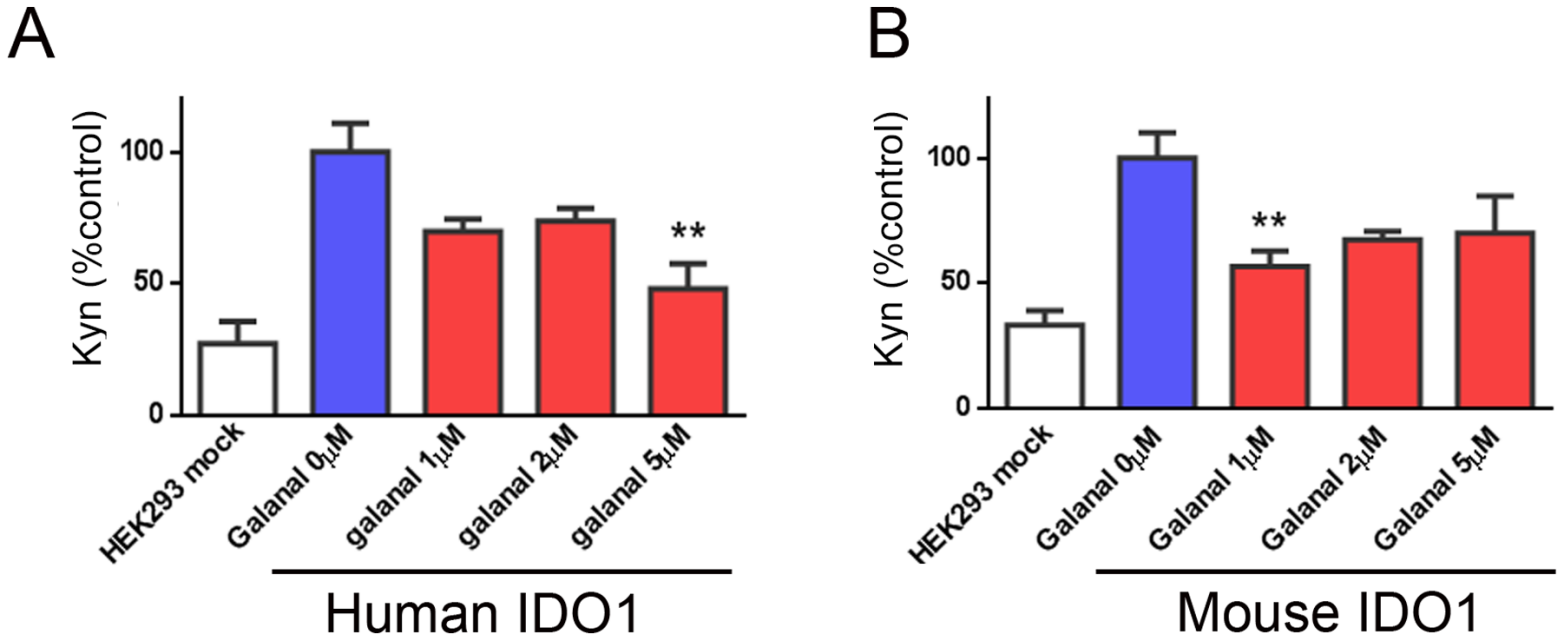
Effect of galanal on IDO1 activity in different cell species. HEK 293 cells transfected with human IDO1 (A) or mouse IDO1 (B) were treated with various concentrations (1-5 µM) of galanal for 48 hrs. Data are mean ± SD (n = 3). **P<0.01.

The inhibitory effect of galanal on IDO1 activity was examined in human IDO1-overexpressed cells and mouse IDO1-overexpressed cells in order to clarify the specificity of the compound on IDO1 species. When human IDO1-overexpressed cells and mouse IDO1-overexpressed cells were treated with various concentrations of galanal (1–5 µM), galanal inhibited both enzymatic activities of human IDO1 and mouse IDO1 in a dose-dependent manner (Figure 2).

### Kinetic analysis for IDO1 inhibition by galanal

**Figure 3:**
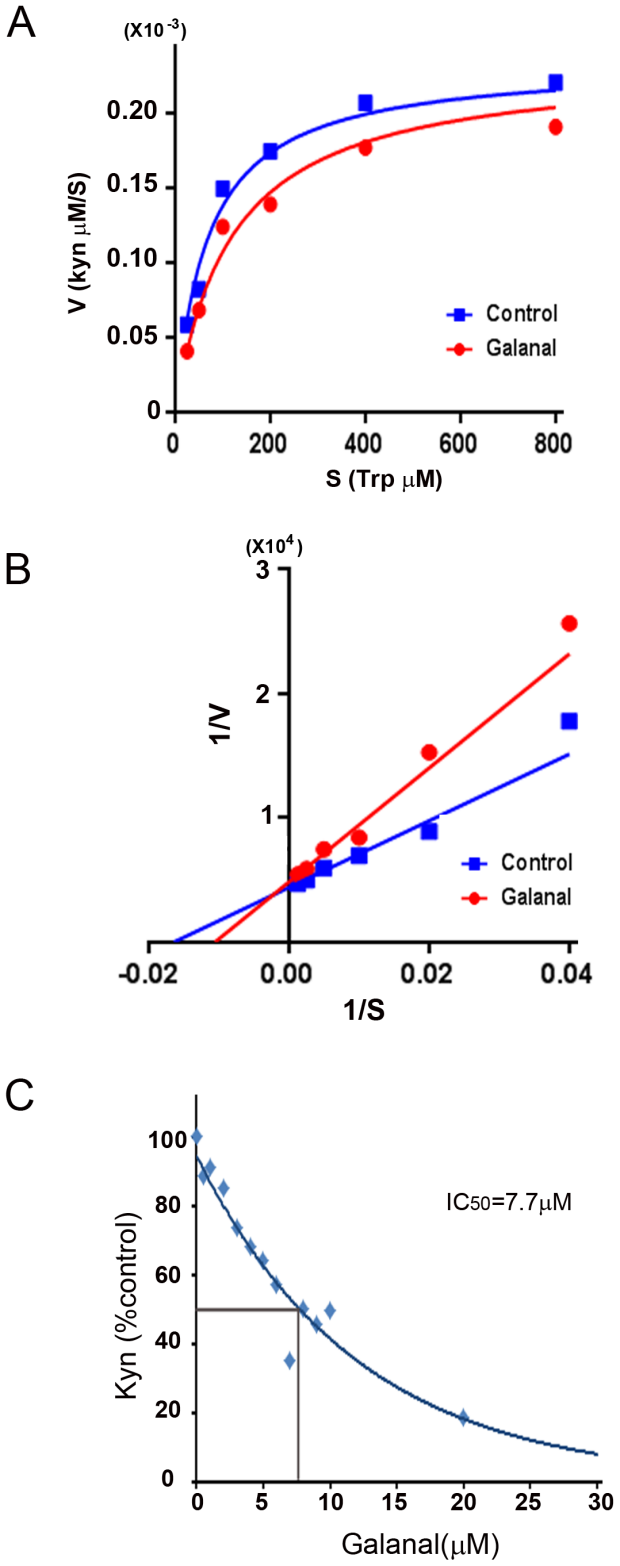
Kinetic analysis for enzymatic IDO1 inhibition by galanal and IC_50_ value of galanal. The mode of enzymatic IDO1 inhibition by galanal. The mode of the inhibition was analyzed using the (A) Michaelis-Menten model, or (B) the Lineweaver-Burk plot in the presence or absence of galanal. (C) The IC_50_ value of galanal for the enzymatic activity of IDO1.

Results of the kinetic studies of IDO1 using two concentrations (0 and 30 µM) of galanal were plotted on the Michaelis–Menten model; galanal lowered the Vmax 0.88 folds determinations when Trp was varied, but it had pronounced effects on the apparent Michaelis constants in Figure 3A (Km = 1/2 Vmax, Control; 52.6 µM, galanal; 74.5 µM).

The mode of IDO1 inhibition by galanal was examined using the Lineweaver-Burk plot (Figure 3B). Galanal inhibited the activity of the purified recombinant human IDO1 with an increase in its concentration and caused an almost complete inhibition of the enzymatic activity at 30 µM; the IC_50_ value of galanal was 7.7 µM, proving that it inhibited IDO1 activity in a competitive manner (Figure 3C).

### Effect of galanal on the NFκ-B signal transduction pathway

**Figure 4:**
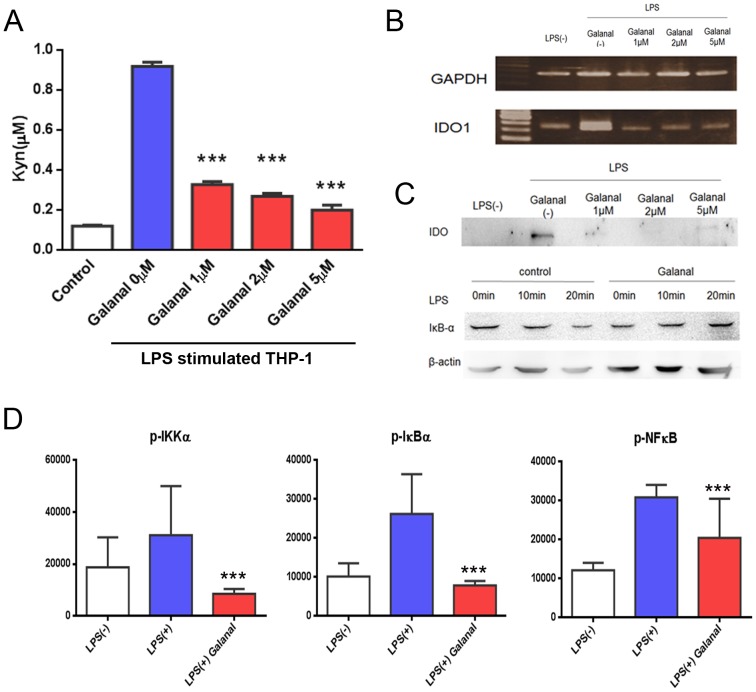
Inhibition by galanal of the NFκ-B signal transduction pathway. (A) L-Kyn formation in LPS (50 ng/ml)-treated THP-1 cells with and without various concentrations (1-5 µM) of galanal. L-Kyn formed after 24-hr incubation was measured by HPLC. Data are the mean ± SD. ***P<0.001. (B) Expression of IDO1 mRNA in LPS-treated THP-1 cells with and without galanal after 24 hrs incubation. The mRNA expressed was measured using RT-PCR. (C) Cytoplasmic expression of IDO1 and Iκ-Bα proteins in LPS (50 ng/ml)-treated THP-1 cells with and without galanal after 24 hrs incubation. (D) The phosphorylation of IKK-α, IκB-α, and NFκ-B in LPS (50 ng/ml)-treated THP-1 cells with and without galanal after 60-min incubation. Data are the mean ± SD. ***P<0.001.

As shown in Figure 4, galanal inhibited IDO1 induction in LPS-stimulated THP-1cells, contingent on the dose. The inhibitory ability of galanal was confirmed by measuring the levels of both L-Kyn production and IDO1 mRNA expression (Figure 4A and B). Galanal decreased the expression of IDO1 mRNA in a dose-dependent manner, suggesting that galanal could inhibit signal transduction by LPS stimulus. It is known that LPS stimulus up-regulates IDO1 expression through the NFκ-B signal transduction pathway [2], [3]. To confirm the effect of galanal on the NFκ-B signal transduction pathway, IκB-α expression and the phosphorylation of IKK-α, IκB-α, and NFκ-B were examined. Galanal did not decrease the IκB-α expression in LPS-stimulated THP-1 cells (Figure 4C). The phosphorylation of IKK-α, IκB-α, and NFκ-B were down-regulated by Galanal (Figure 4D). Galanal was thus found to interfere with the transcriptional function of the NFκ-B signaling pathway by reducing the degradation of signal transducer IκB-α.

### Effect of galanal on the IFN-γ signal transduction pathway

**Figure 5:**
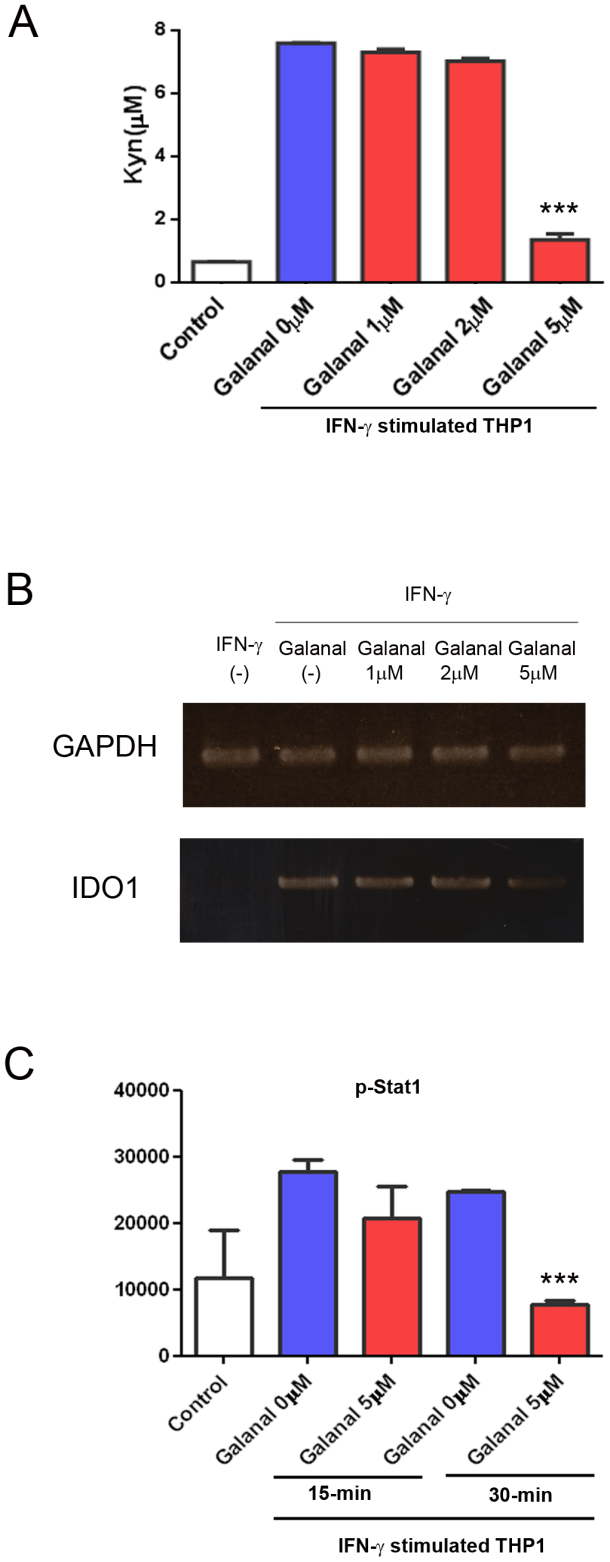
Inhibition by galanal of the IFN-γ dependent pathway. (A) L-Kyn formation in IFN-γ (100U)-treated THP-1 cells with and without various concentrations (1-5 µM) of galanal. L-Kyn formed after 24-hr incubation was measured by HPLC. Data are the mean ± SD. ***P<0.001. (B) Expression of IDO1 mRNA in IFN-γ (100 U)-treated THP-1 cells with and without galanal (1-5 µM) after 24-hr incubation. IDO1 mRNA expressed in IFN-γ -treated THP-1 cells with and without galanal was measured using RT-PCR. (C) The phosphorylation of stat1 in IFN-γ (100 U)-treated THP-1 cells with and without galanal after 15-min or 30-min incubation. n. Data are the mean ± SD. ***P<0.001.

Galanal inhibited both L-Kyn production and IDO1 mRNA induction in IFN-γ-stimulated THP-1cells (Figure 5A and B). The phosphorylation of stat1 was down-regulated by Galanal (Figure 5C). Furthermore, galanal interfered with the transcriptional function of the IFN-γ dependent pathway.

### Inhibition of cellular IDO1 activity by galanal

**Figure 6:**
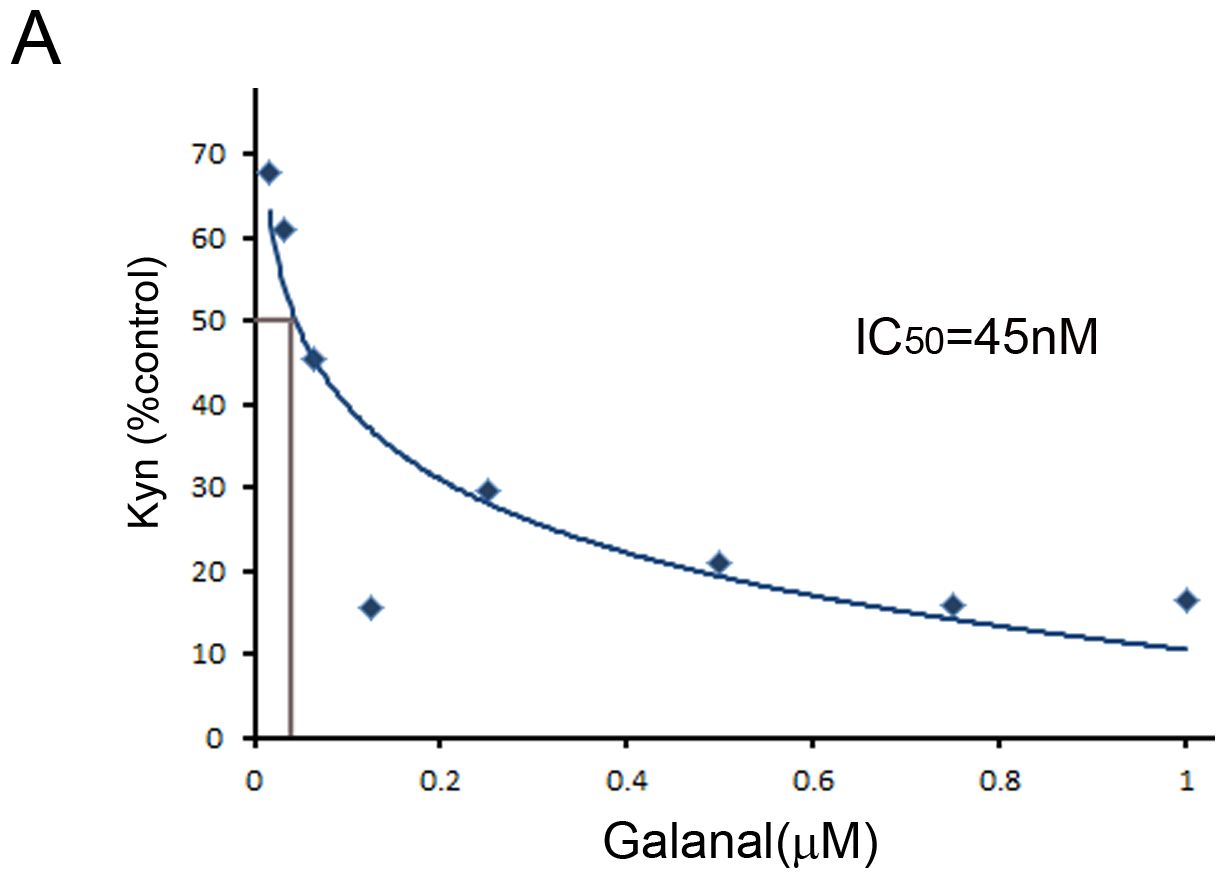
Inhibition of cellular IDO1 activity by galanal. LPS-stimulated THP-1 cells were exposed to various concentrations of galanal. Percentage inhibition in LPS-stimulated THP-1 cells against control cells was plotted and IC_50_ value determined.

Cellular IDO1 inhibition by galanal was examined in LPS-stimulated THP-1 cells. As shown in Figure 6, galanal caused a clear inhibition of the cellular IDO1 activity and when the percentage of inhibition of IDO1 activity by galanal in LPS-stimulated THP-1 cells against control cells was plotted, the IC_50_ value was found to be 45 nM.

## Discussion

IDO1 is recognized as one of the prominent mediators of immune regulation by metabolic pathways. Therefore, the regulation of the enzymatic activity of IDO1 is useful in the treatments of tumor, inflammatory diseases, and depression. Previous data have suggested that IDO1 inhibition can modulate immune response, delay tumor growth, and enhance dendritic cell vaccines [21] [22]. A frequently used IDO1 inhibitor is 1-methyl-tryptophan (1-MT). Several studies have provided evidence that IDO1 inhibition with 1-MT or other small molecule inhibitors can exert antitumor effects [23] [24]. Initial evidence, in 2002, showed that IDO1 inhibitor 1MT could partly retard growth of mouse melanoma cells engrafted onto a syngeneic host [25]. A recent report showed that 1-MT with an anticancer drug, improved tumor prognosis [26]. It seems that D-1-methyl-tryptophan (D-1-MT) selected for phase I trials has immune modulating activity in the United States [24]. However, some reports have shown that D-1-MT inhibits IDO2, a recently identified IDO1 isoform, more selectively than IDO1 [27] [28] [29]. The mechanisms by which D-1-MT inhibits IDO1 and IDO2 have yet to be cleared.

Considerable attention has been paid to the research of effective IDO1 inhibitors [30] [31] [32]. The IDO1 inhibitor search was based on indole-based structures, combination of docking-based pharmacophore model development, and several natural products. Among the IDO1 inhibitors studied so far, it was seen that phytochemicals such as EGCG and curcumin inhibited the activity of IDO1 [15], [16]. To find a new, effective IDO1 inhibitor, we examined the ability of various phytochemicals to inhibit IDO1 activity: galanal was found to have a strong ability to inhibit IDO1 activity in a competitive manner. Galanal is extracted from Myoga, a perennial herb with pungently aromatic flower buds, native to Eastern Asia. In Japan, the flower buds of Myoga are used as a spice and eaten as pickles. The labdane-type diterpene trialdehydes, 12(E)-lab-dene-15,16,(8β),17-trial(miogatrial), galanal A, and galanal B, are found in Myoga flower buds and exhibit certain biological activities[17] [33].

Though many inhibitors for IDO1 have been found, there are few inhibitors with competitive inhibition. In the present study, galanal was found to possess more IDO1 inhibitory potency than either the most-commonly used 1-MT or other previously-published inhibitors (Table. 1) [14] [28] [34] [35] [36] [37] [38] [39] [40] [41] [42] [43]. Since the IC_50_ value of inhibitor depends on the IDO1 enzyme activity in each experiment, we could not unconditionally compare the previous results. However, these results showed that galanal is an effective IDO1 inhibitor because a very low dosage of it showed inhibitory activity in the cell-based assay.

**Table 1:** The list of published IDO1 inhibitor.

Inhibitor	IC50	Method	Reference
Galanal	7.7 µM	recombinant humanIDO1	
	45nM	THP-1 cell assay	
1-Methyl-DL-Tryptophan	380 µM	recombinant humanIDO1	Cady SG et al. (1991)
6-Chloro-DL-Tryptophan	51 µM	THP-1cell assay	Saito K et al. (1993)
Norharman	43 µM	THP-1cell assay	Saito K et al. (1993)
Brassinin	37.9 µM	COS-1 cell assay(human IDO1)	Banerjee T et al. (2008)
	31.1 µM	COS-1 cell assay(mouse IDO1)	Banerjee T et al. (2008)
5-Bromo-Brassinin	24 µM	COS-1 cell assay(human IDO1)	Banerjee T et al. (2008)
	26.1 µM	COS-1 cell assay(mouse IDO1)	Banerjee T et al. (2008)
Menadione	28.9 µM	T-RE-derived cell assay	Kumar S et al. (2008)
4-Amino-1,2,5-Oxadiazole-3-Carboximidamide	1 µM	HeLa cell assay	Yue EW et al. (2009)
1-Ethyl-Tryptophan	100 µM	mouse DC cell assay	Sun T et al (2010)
INCB024360	10 nM	HeLa cell assay	Liu X et al. (2010)
	>30 µM	THP-1 cell assay	Liu X et al. (2010)
S-Benzylisothiourea Derivatives	0.1 µM	A431 cell assay	Matsuno K et al. (2010)
4-Aryl-1H-1,2,3-Triazole	86 µM	recombinant humanIDO1	Huang Q et al. (2011)
Amg-1	3 µM	Briedge-IT Try fluorescence assay	Meininger D et al. (2011)
MTH-Tryptophan	82.5 µM	Briedge-IT Try fluorescence assay	Meininger D et al. (2011)
N-Methyl-L-Tryptophan	35.6 µM	Briedge-IT Try fluorescence assay	Meininger D et al. (2011)
β-Lapachone	440 nM	recombinant humanIDO1	Flick HE et al (2013)
	1 µM	HeLa cell assay	Flick HE et al (2013)
Pyranonaphthoquinone derivates	6 µM	recombinant humanIDO1	Bridewell DJA et al. (2013)
	5.4 µM	LLTC cell assay	Bridewell DJA et al. (2013)

IDO1 is known to be induced by both the IFN-γ -dependent pathway and the IFN-γ-independent pathway, i.e., the NF-κB-dependent pathway [2] [3]. Galanal inhibited not only the enzymatic activity of IDO1 but also suppressed the signal transduction associated with the expression of IDO1 mRNA. Namely, a very low dose of galanal inhibited the expression of IDO1 mRNA induced by the NF-κB-dependent pathway clearly. In addition, it was suggested that galanal could inhibit the expression of IDO1 mRNA induced by the IFN-γ -dependent pathway. NFκ-B is activated by various stimuli such as free radicals, inflammatory stimuli, cytokines, carcinogens, tumor promoters, endotoxins, radiation, and ultraviolet light [44]. Upon activation, NFκ-B induces the expression of more than 200 genes known to suppress apoptosis and also induces cellular transformation, proliferation, invasion, metastasis, chemoresistance, radio-resistance, and inflammation [45]. Many of the target genes that are activated are critical to the establishment of inflammation and tumor [46]. Therefore, control of the NFκ-B signal transduction may be useful for treatments of tumor and inflammatory diseases.

Our results have shown that galanal is an effective compound in immunomodulation, because the compound inhibits both the certain signal transduction and enzymatic activity of IDO1 at a very low dose.

## Conclusion

The results of the present study indicate that galanal is a competitive IDO1 inhibitor. The results also indicate that a very low dose of galanal inhibits not only the enzymatic activity of IDO1 but also the expression of IDO1 mRNA induced by the NF-κB-dependent pathway. Furthermore, the results suggest that galanal could inhibit the expression of IDO1 mRNA induced by the IFN-γ -dependent pathway. Therefore, galanal and its derivatives may be useful for the treatment of inflammatory diseases.

## References

[1] Hassanain HH, Chon SY, Gupta SL (1993) Differential regulation of human indoleamine 2,3-dioxygenase gene-expression by interferon-gamma and interferon-alpha-analysis of the regulatory region of the gene and identification of an interferon-gamma-inducible DNA-binding factor. Journal of Biological Chemistry 268: 5077–5084. 8444884

[2] Fujigaki H, Saito K, Fujigaki S, Takemura M, Sudo K, et al (2006) The signal transducer and activator of transcription 1 alpha and interferon regulatory factor 1 are not essential for the induction of indoleamine 2,3-dioxygenase by lipopolysaccharide: Involvement of p38 mitogen-activated protein kinase and nuclear Factor-kappa B pathways, and synergistic effect of several proinflammatory cytokines. Journal of Biochemistry 139: 655–662. 16672265 10.1093/jb/mvj072

[3] Fujigaki S, Saito K, Sekikawa K, Tone S, Takikawa O, et al (2001) Lipopolysaccharide induction of indoleamine 2,3-dioxygenase is mediated dominantly by an IFN-gamma-independent mechanism. European Journal of Immunology 31: 2313–2318. 11477543 10.1002/1521-4141(200108)31:8<2313::aid-immu2313>3.0.co;2-s

[4] Frumento G, Rotondo R, Tonetti M, Damonte G, Benatti U, et al (2002) Tryptophan-derived catabolites are responsible for inhibition of T and natural killer cell proliferation induced by indoleamine 2,3-dioxygenase. Journal of Experimental Medicine 196: 459–468. 12186838 10.1084/jem.20020121 PMC2196046

[5] Sharma MD, Hou D-Y, Liu Y, Koni PA, Metz R, et al (2009) Indoleamine 2,3-dioxygenase controls conversion of Foxp3(+) Tregs to TH17-like cells in tumor-draining lymph nodes. Blood 113: 6102–6111. 19366986 10.1182/blood-2008-12-195354 PMC2699232

[6] Grohmann U, Orabona C, Fallarino F, Vacca C, Calcinaro F, et al (2002) CTLA-4-Ig regulates tryptophan catabolism in vivo. Nature Immunology 3: 1097–1101. 12368911 10.1038/ni846

[7] Munn DH, Zhou M, Attwood JT, Bondarev I, Conway SJ, et al (1998) Prevention of allogeneic fetal rejection by tryptophan catabolism. Science 281: 1191–1193. 9712583 10.1126/science.281.5380.1191

[8] Palafox D, Llorente L, Alberu J, Torres-Machorro A, Camorlinga N, et al (2010) The role of indoleamine 2,3 dioxygenase in the induction of immune tolerance in organ transplantation. Transplantation Reviews 24: 160–165. 20541386 10.1016/j.trre.2010.04.003

[9] Boasso A, Vaccari M, Fuchs D, Hardy AW, Tsai W-P, et al (2009) HIV-induced tryptophan catabolism: pathogenic mechanism and target for immunotherapy. Amino Acids 37: 89–89.18683019

[10] Romani L, Fallarino F, De Luca A, Montagnoli C, D'Angelo C, et al (2008) Defective tryptophan catabolism underlies inflammation in mouse chronic granulomatous disease. Nature 451: 211–U212. 18185592 10.1038/nature06471

[11] Platten M, Ho PP, Youssef S, Fontoura P, Garren H, et al (2005) Treatment of autoimmune neuroinflammation with a synthetic tryptophan metabolite. Science 310: 850–855. 16272121 10.1126/science.1117634

[12] Myint AM, Schwarz MJ, Muller N (2012) The role of the kynurenine metabolism in major depression. Journal of Neural Transmission 119: 245–251. 22139324 10.1007/s00702-011-0741-3

[13] Muller AJ, Scherle PA (2006) Targeting the mechanisms of tumoral immune tolerance with small-molecule inhibitors. Nature Reviews Cancer 6: 613–625. 16862192 10.1038/nrc1929

[14] Bridewell DJA, Sperry J, Smith JR, Kosim-Satyaputra P, Ching L-M, et al (2013) Natural Product-Inspired Pyranonaphthoquinone Inhibitors of Indoleamine 2,3-Dioxygenase-1 (IDO-1). Australian Journal of Chemistry 66: 40–49.

[15] Jeong Y-I, Jung ID, Lee JS, Lee C-M, Lee J-D, et al. (2007) (-)-Epigallocatechin gallate suppresses indoleamine 2,3-dioxygenase expression in murine dendritic cells: Evidences for the COX-2 and STAT1 as potential targets. Biochemical and Biophysical Research Communications 354.. 10.1016/j.bbrc.2007.01.076 17270146

[16] Jeong Y-I, Kim SW, Jung ID, Lee JS, Chang JH, et al. (2009) Curcumin Suppresses the Induction of Indoleamine 2,3-Dioxygenase by Blocking the Janus-activated Kinase-Protein Kinase C delta-STAT1 Signaling Pathway in Interferon-gamma-stimulated Murine Dendritic Cells. Journal of Biological Chemistry 284.. 10.1074/jbc.M807328200 19075017

[17] Abe M, Ozawa Y, Uda Y, Yamada Y, Morimitsu Y, et al (2002) Labdane-type diterpene dialdehyde, pungent principle of myoga, Zingiber mioga Roscoe. Bioscience Biotechnology and Biochemistry 66: 2698–2700. 10.1271/bbb.66.2698 12596870

[18] Hoshi M, Ito H, Fujigaki H, Takemura M, Takahashi T, et al (2009) Indoleamine 2,3-dioxygenase is highly expressed in human adult T-cell leukemia/lymphoma and chemotherapy changes tryptophan catabolism in serum and reduced activity. Leuk Res 33: 39–45. 18639341 10.1016/j.leukres.2008.05.023

[19] Fujigaki S, Saito K, Takemura M, Fujii H, Wada H, et al (1998) Species differences in L-tryptophan-kynurenine pathway metabolism: quantification of anthranilic acid and its related enzymes. Arch Biochem Biophys 358: 329–335. 9784247 10.1006/abbi.1998.0861

[20] Fujigaki H, Saito K, Lin F, Fujigaki S, Takahashi K, et al (2006) Nitration and inactivation of IDO by peroxynitrite. Journal of Immunology 176: 372–379. 10.4049/jimmunol.176.1.372 16365430

[21] Prendergast GC, Metz R Novel isolated indoleamine 2,3-dioxygenase-2 protein, useful for treating melanoma, hepatitis C viral infection, prion disease, metabolic disorders, cardiovascular disease, age related disorders and neurological disorders. Lankenau Inst Medical Res; Prendergast G C; Metz R.

[22] Uyttenhove C, Pilotte L, Theate I, Stroobant V, Colau D, et al (2003) Evidence for a tumoral immune resistance mechanism based on tryptophan degradation by indoleamine 2,3-dioxygenase. Nature Medicine 9: 1269–1274. 10.1038/nm934 14502282

[23] Liu X, Newton RC, Friedman SM, Scherle PA (2009) Indoleamine 2,3-Dioxygenase, an Emerging Target for Anti-Cancer Therapy. Current Cancer Drug Targets 9: 938–952. 20025603 10.2174/156800909790192374

[24] Soliman H, Mediavilla-Varela M, Antonia S (2010) Indoleamine 2,3-Dioxygenase Is It an Immune Suppressor? Cancer Journal 16: 354–359. 10.1097/PPO.0b013e3181eb3343 PMC3850167 20693847

[25] Munn DH, Sharma MD, Hou D, Baban B, Lee JR, et al (2004) Expression of indoleamine 2,3-dioxygenase by plasmacytoid dendritic cells in tumor-draining lymph nodes. Journal of Clinical Investigation 114: 280–290. 15254595 10.1172/JCI21583 PMC449750

[26] Wang D, Saga Y, Mizukami H, Sato N, Nonaka H, et al (2012) Indoleamine-2,3-dioxygenase, an immunosuppressive enzyme that inhibits natural killer cell function, as a useful target for ovarian cancer therapy. International Journal of Oncology 40: 929–934. 22179492 10.3892/ijo.2011.1295 PMC3584823

[27] Loeb S, Koenigsrainer A, Zieker D, Bruecher BLDM, Rammensee H-G, et al (2009) IDO1 and IDO2 are expressed in human tumors: levo- but not dextro-1-methyl tryptophan inhibits tryptophan catabolism. Cancer Immunology Immunotherapy 58: 153–157. 18418598 10.1007/s00262-008-0513-6 PMC11030193

[28] Meininger D, Zalameda L, Liu Y, Stepan LP, Borges L, et al (2011) Purification and kinetic characterization of human indoleamine 2,3-dioxygenases 1 and 2 (IDO1 and IDO2) and discovery of selective IDO1 inhibitors. Biochimica Et Biophysica Acta-Proteins and Proteomics 1814: 1947–1954. 10.1016/j.bbapap.2011.07.023 21835273

[29] Hou D-Y, Muller AJ, Sharma MD, DuHadaway J, Banerjee T, et al (2007) Inhibition of indoleamine 2,3-dioxygenase in dendritic cells by stereoisomers of 1-methyl-tryptophan correlates with antitumor responses. Cancer Research 67: 792–801. 17234791 10.1158/0008-5472.CAN-06-2925

[30] Cerejo M, Andrade G, Roca C, Sousa J, Rodrigues C, et al (2012) A Powerful Yeast-Based Screening Assay for the Identification of Inhibitors of Indoleamine 2,3-Dioxygenase. Journal of Biomolecular Screening 17: 1362–1371. 22791376 10.1177/1087057112452595

[31] Lancellotti S, Novarese L, De Cristofaro R (2011) Biochemical Properties of Indoleamine 2,3-dioxygenase: From Structure to Optimized Design of Inhibitors. Current Medicinal Chemistry 18: 2205–2214. 21517759 10.2174/092986711795656108

[32] Smith JR, Evans KJ, Wright A, Willows RD, Jamie JF, et al (2012) Novel indoleamine 2,3-dioxygenase-1 inhibitors from a multistep in silico screen. Bioorganic & Medicinal Chemistry 20: 1354–1363. 22112538 10.1016/j.bmc.2011.10.068

[33] Abe M, Ozawa Y, Morimitsu Y, Kubota K (2008) Mioganal, a Novel Pungent Principle in Myoga (Zingiber mioga Roscoe) and a Quantitative Evaluation of Its Pungency. Bioscience Biotechnology and Biochemistry 72: 2681–2686. 10.1271/bbb.80347 18838802

[34] Sun T, Chen X-H, Tang Z-D, Cai J, Wang X-Y, et al (2010) Novel 1-alkyl-tryptophan derivatives downregulate IDO1 and IDO2 mRNA expression induced by interferon-gamma in dendritic cells. Molecular and Cellular Biochemistry 342: 29–34. 20424892 10.1007/s11010-010-0465-y

[35] Saito K, Chen CY, Masana M, Crowley JS, Markey SP, et al (1993) 4-Chloro-3-hydroxyanthranilate, 6-chlorotryptophan and norharmane attenuate quinolinic acid formation by interferon-gamma-stimulated monocytes (THP-1 Cells). Biochemical Journal 291: 11–14. 8471029 10.1042/bj2910011 PMC1132472

[36] Liu X, Shin N, Koblish HK, Yang G, Wang Q, et al (2010) Selective inhibition of IDO1 effectively regulates mediators of antitumor immunity. Blood 115: 3520–3530. 20197554 10.1182/blood-2009-09-246124

[37] Kumar S, Malachowski WP, DuHadaway JB, LaLonde JM, Carroll PJ, et al (2008) Indoleamine 2,3-dioxygenase is the anticancer target for a novel series of potent naphthoquinone-based inhibitors (vol 51, pg 1712, 2008). Journal of Medicinal Chemistry 51: 7325–7325. 10.1021/jm7014155 PMC4384695 18318466

[38] Banerjee T, DuHadaway JB, Gaspari P, Sutanto-Ward E, Munn DH, et al (2008) A key in vivo antitumor mechanism of action of natural product-based brassinins is inhibition of indoleamine 2,3-dioxygenase. Oncogene 27: 2851–2857. 18026137 10.1038/sj.onc.1210939

[39] Matsuno K, Takai K, Isaka Y, Unno Y, Sato M, et al (2010) S-Benzylisothiourea derivatives as small-molecule inhibitors of indoleamine-2,3-dioxygenase. Bioorganic & Medicinal Chemistry Letters 20: 5126–5129. 20688518 10.1016/j.bmcl.2010.07.025

[40] Yue EW, Douty B, Wayland B, Bower M, Liu X, et al (2009) Discovery of Potent Competitive Inhibitors of Indoleamine 2,3-Dioxygenase with in Vivo Pharmacodynamic Activity and Efficacy in a Mouse Melanoma Model. Journal of Medicinal Chemistry 52: 7364–7367. 19507862 10.1021/jm900518f

[41] Huang Q, Zheng M, Yang S, Kuang C, Yu C, et al (2011) Structure-activity relationship and enzyme kinetic studies on 4-aryl-1H-1,2, 3-triazoles as indoleamine 2,3-dioxygenase (IDO) inhibitors. European Journal of Medicinal Chemistry 46: 5680–5687. 21925773 10.1016/j.ejmech.2011.08.044

[42] Cady SG, Sono M (1991) 1-Methyl-DL-tryptophan, beta-(3-benzofuranyl)-DL-alanine (the oxygen analog of tryptophan), and beta- 3-benzo(b)thienyl-DL-alanine (the sulfur analog of tryptophan) are competitive inhibitors for indoleamine 2,3-dioxygenase. Archives of Biochemistry and Biophysics 291: 326–333. 1952947 10.1016/0003-9861(91)90142-6

[43] Banerjee T, DuHadaway JB, Gaspari P, Sutanto-Ward E, Munn DH, et al (2013) A key in vivo antitumor mechanism of action of natural product-based brassinins is inhibition of indoleamine 2,3-dioxygenase. Oncogene 27(20): 2851–7. 10.1038/sj.onc.1210939 18026137

[44] Kumar A, Takada Y, Boriek AM, Aggarwal BB (2004) Nuclear factor-kappa B: its role in health and disease. Journal of Molecular Medicine-Jmm 82: 434–448. 10.1007/s00109-004-0555-y 15175863

[45] Nakanishi C, Toi M (2005) Nuclear factor-kappa B inhibitors as sensitizers to anticancer drugs. Nature Reviews Cancer 5: 297–309. 15803156 10.1038/nrc1588

[46] Pahl HL (1999) Activators and target genes of Rel/NF-kappa B transcription factors. Oncogene 18: 6853–6866. 10602461 10.1038/sj.onc.1203239

